# “Script Killing” immersive teaching improves the competency of emergency medicine residents rotating across departments

**DOI:** 10.3389/fpubh.2026.1806065

**Published:** 2026-03-19

**Authors:** Dalong Zhang, Jiankai Gao, Xingguo Niu, Haoran Si, Yang Sun, Mingzhe Li, Tianyi Yao, Juhua Tang

**Affiliations:** 1 The Fifth Clinical Medical College of Henan University of Chinese Medicine (Zhengzhou People's Hospital), Zhengzhou, China; 2 Henan University of Chinese Medicine, Zhengzhou, China

**Keywords:** “Script Killing”, clinical competence, emergency medicine, immersive teaching, medical education innovation, standardized residency training

## Abstract

**Objective:**

To evaluate whether “Script Killing” immersive teaching improves job competency among resident physicians rotating through the emergency department across departments.

**Methods:**

We conducted a cohort-allocated controlled educational study at Zhengzhou People’s Hospital (November 2024 to March 2025). Thirty-six rotating residents were allocated by cohort to an intervention group (*n* = 18; “Script Killing” immersive role-play, also referred to as a murder-mystery game–based scenario teaching) or a control group (*n* = 18; traditional teaching). Outcomes included written theoretical assessment, objective structured clinical examination (OSCE), teaching satisfaction, recognition of the teaching process, clinical decision-making indicators (e.g., triage accuracy and reasonableness of emergency management plans), and 3-month knowledge retention.

**Results:**

After 3 months, the intervention group scored higher than controls in theoretical knowledge (86.82 ± 2.85 vs. 72.45 ± 3.25) and OSCE performance (92.47 ± 3.22 vs. 75.18 ± 4.08) (both *p* < 0.001). Triage accuracy was higher in the intervention group (94.44% vs. 66.67%, *p* = 0.034). The reasonableness of management plans was numerically higher but did not reach statistical significance (88.89% vs. 61.11%, *p* = 0.054). At 3-month follow-up, the intervention group showed better knowledge retention, with smaller declines in theoretical and OSCE scores from baseline (*p* < 0.001).

**Conclusion:**

“Script Killing” immersive teaching was associated with improved examination performance, OSCE skills, and key decision-making behaviors, with higher learner satisfaction and improved knowledge retention among cross-department rotating residents in the emergency department.

## 1 Introduction

As the forefront of medical treatment, emergency medicine is characterized by high intensity, high timeliness, and multidisciplinary intersection. According to the “Contents and Standards for Standardized Residency Training (2022 Edition),” resident physicians need to master the whole-chain clinical skills through cross-departmental rotations. However, the on-the-job competency attainment rate is low when non-emergency specialist physicians rotate to the emergency department (1), mainly due to three major bottlenecks in traditional teaching: (1) Scenario fragmentation: PPT lectures and case discussions are difficult to simulate the multitasking pressure of the emergency department (e.g., cardiopulmonary resuscitation synchronized with family communication) (2); (2) Superficial collaboration: PBL/TBL and other models have blurred team divisions and lack role assignment, making it difficult to train genuine collaborative effectiveness; and (3) Limited error tolerance: High-risk procedures (such as tracheal intubation) cannot be deeply trained in a real environment.

While simulation-based teaching can partially alleviate the aforementioned problems, issues such as low engagement and insufficient interprofessional integration remain significant. In recent years, “Script Killing” have demonstrated innovative potential in the field of medical education due to their advantages of strong immersion (role-playing), high interactivity (chain-task driven), and dynamic intervention mechanisms (NPCs creating real-time variables). Its core value lies in: (1) Restoring the entire emergency process: By designing the scenario of “pre-hospital emergency care—in-hospital resuscitation—ward handover” (3), it breaks the scene fragmentation of traditional teaching. (2) Assigned roles: Clearly defined hierarchical tasks (e.g., junior nurse → senior resuscitation commander) to avoid the “bystander effect” in teamwork (4); and (3) Zero-risk trial and error: NPC simulates medical errors (such as family members refusing to sign), accelerating experience accumulation.

However, current studies mostly focus on the application in a single department, lacking controlled validation for the interdepartmental rotation group. To address this gap, we implemented “Script Killing” immersive teaching in emergency rotation training for non-emergency residents and evaluated its educational impact using a cohort-allocated controlled design to minimize contamination within the same cohort. The intervention incorporated full-process scenario construction and role-based task progression, covering core emergency competencies (e.g., airway management and mass casualty response). Using a multidimensional evaluation framework (knowledge tests, OSCE performance, satisfaction and process recognition, and behavioral decision-making indicators), we examined whether immersive scenario-based learning could improve emergency response capability and interprofessional collaboration among rotating residents.

## 2 Research subjects and methods

### 2.1 Research subjects

According to the “Standardized Training Content and Standards for Resident Physicians” (2022 Edition), resident trainees from all departments undergo parallel rotations across multiple departments, with most rotating through the emergency department. This study selected 36 resident physicians from different cohorts (same cohort received the same teaching method) undergoing emergency department rotations at Zhengzhou People’s Hospital between November 2024 and March 2025. They were divided into a control group and an observation group based on the teaching method used. Inclusion criteria: (1) Voluntary participation in this study; (2) Participants agreed to the teaching methods used; and (3) Detailed records of emergency medicine teaching and evaluation data were available. Exclusion criteria: (1) Failure to complete the entire emergency department rotation; (2) Personnel transfers preventing completion of follow-up research; and (3) Individuals on external study or further training (5). The control group consisted of 18 physicians (8 male, 10 female), aged 23–33 years, average (25.71 ± 2.50) years. The observation group consisted of 18 physicians (9 male, 9 female), aged 24–32 years, average (25.68 ± 2.47) years. There was no statistically significant difference in baseline characteristics between the two groups (*p* > 0.05), indicating comparability. Allocation was performed at the cohort level (i.e., all residents within the same cohort received the same teaching method) to reduce cross-group contamination during shared rotations and learning activities.

### 2.2 Research methods

#### 2.2.1 Control group

**Figure 1:**
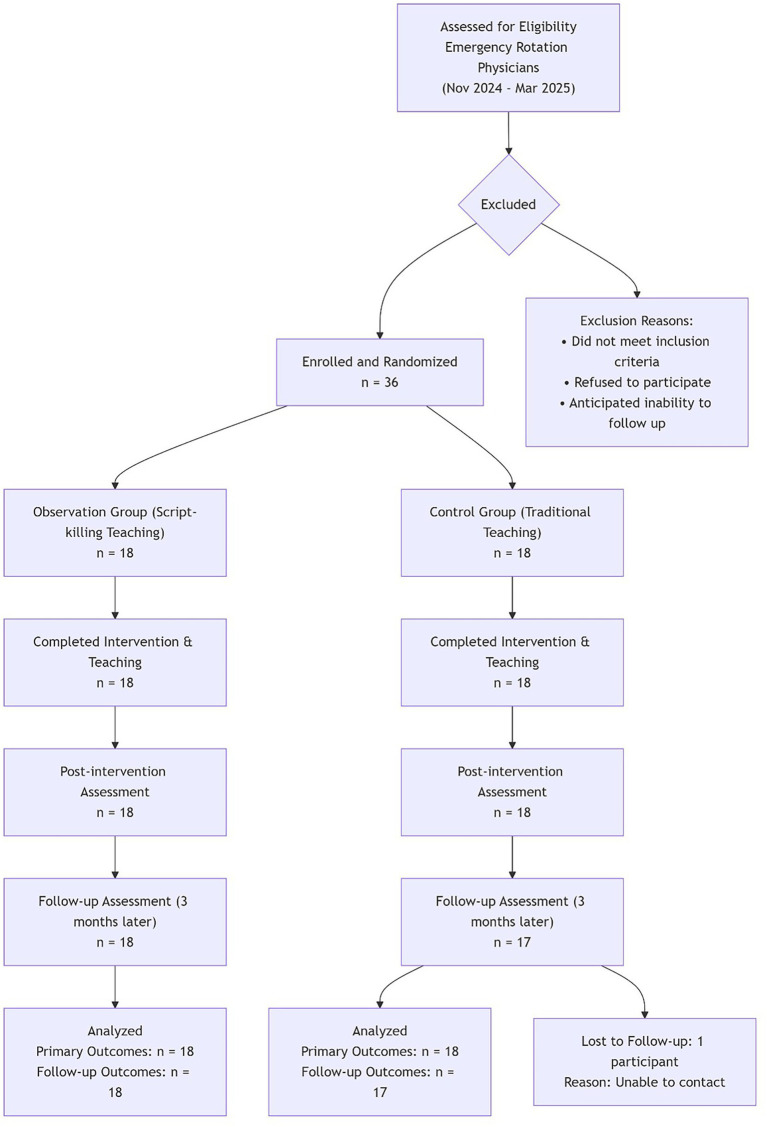
Flow of participants in a cohort-allocated controlled educational intervention.

Emergency medicine teaching was conducted using the traditional teaching model. Based on emergency medicine learning requirements and the teaching syllabus, a teaching plan was developed. Teaching was completed through a combination of theoretical knowledge lectures and practical teaching. Instructors were responsible for explaining and analyzing emergency medicine knowledge. Trainees from different departments completed professional training through methods like note-taking and group discussions (6) (Figure 1).

#### 2.2.2 Observation group

**Figure 2:**
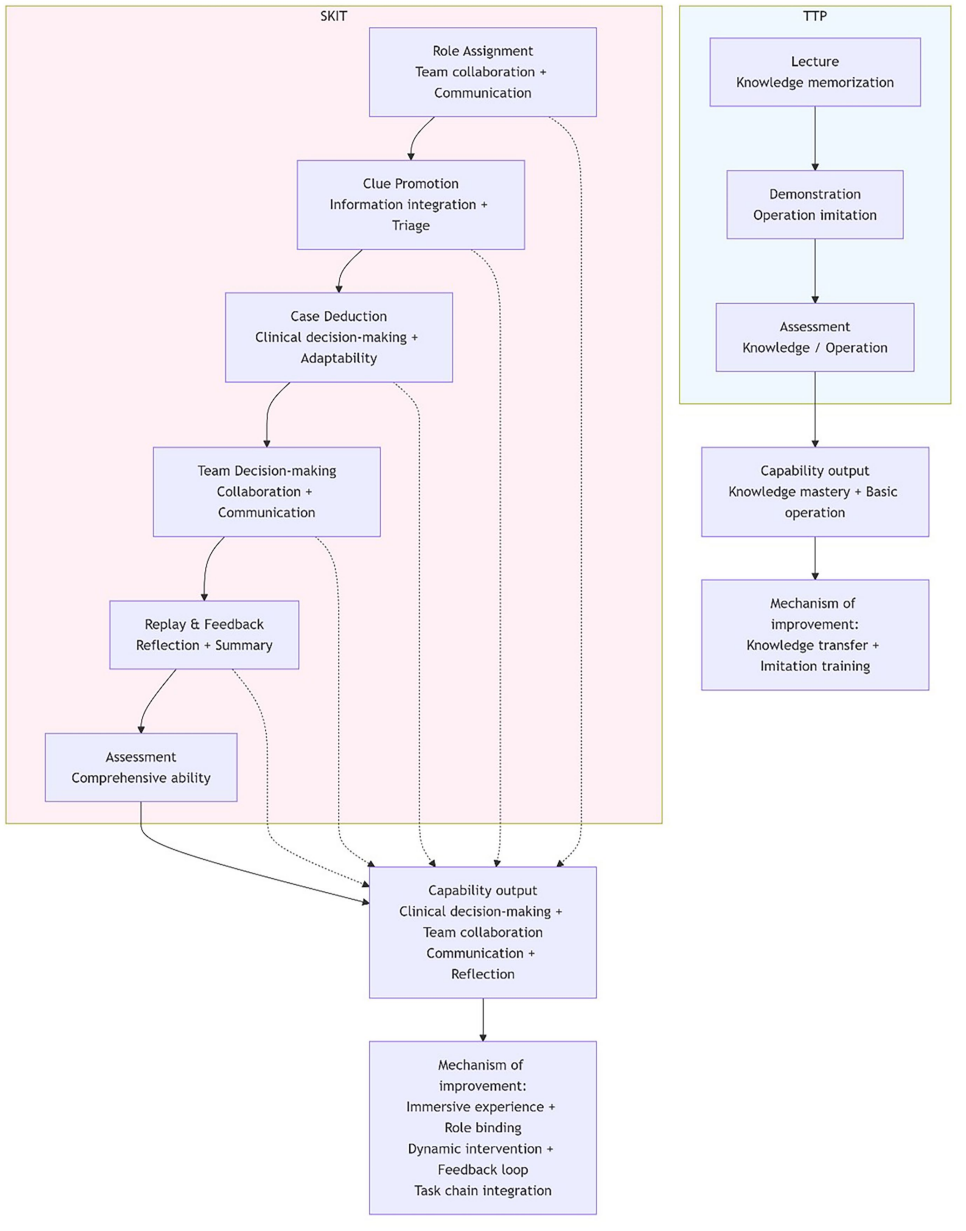
Intervention protocol schematic. TTP: traditional teaching process – knowledge-driven, instructor-led linear pedagogy focusing on knowledge transfer and skill demonstration. SKIT: Script Killing immersive teaching process – scenario-based, role-play immersive pedagogy that enhances clinical decision-making, teamwork, communication, and reflective practice through dynamic case progression and structured feedback.

This group received emergency medicine teaching using Script Killing Immersive Teaching (SKIT), a murder-mystery game–based, role-play, scenario simulation approach. Resident physicians rotating through the emergency department played roles such as emergency department patients, patient family members, doctors, and nurses. The “scripts” were compiled by a writing group composed of experienced teaching physicians. After repeated discussion and selection, representative, typical, and professional emergency department case scripts were finalized. These scripts also presented a certain level of difficulty in management and required communication skills. The scripts detailed factors such as event background, character identities, location settings, scene atmosphere, and specific language/tone (7). Under the guidance of emergency department instructors, deep learning of typical emergency cases was completed. This allowed resident physicians to “participate” in the entire process of patient management in the emergency department, from admission to diagnosis, treatment, and discharge (Figure 2).

The teaching duration corresponded to the rotation period of physicians from different departments in the emergency department, typically 1–3 months. To ensure sufficient time for teaching and research, this study selected resident physicians undertaking a 3-month emergency department rotation as the research subjects. Assessments and evaluations were conducted after teaching completion. The SKIT workflow is illustrated in Box 1.

BOX 1 **Example SKIT scenario (traffic accident with suspected TBI)**

To help readers who are unfamiliar with Script Killing Immersive Teaching (SKIT) understand what occurs during a session, we provide an illustrative scenario. This example is intended to concretize the “script-based, role-play, full-process scenario construction and role-based task progression under instructor guidance” described in the Methods, and does not change the study design or outcome definitions.

**Scenario setting**

Location: ED triage area → resuscitation bay → imaging pathway (head CT) → consultation/transfer handoff.

Background: An adult RTA casualty with impaired consciousness and suspected severe TBI is brought to the ED by prehospital EMS during evening rush hour. Staged information delivery simulates real emergency care challenges: time pressure, uncertainty, incomplete on-scene reports, and unknown medical/medication history.

**Teaching materials**

The learner-facing script specifies the case background, role identities, scenario layout, and task flow, embedding core challenges in clinical operations and doctor–patient communication. Key information is released in a staged manner under instructor facilitation (e.g., using facilitator cue prompts such as “clue cards” and teaching guides) and is provided only after learners complete targeted actions (e.g., focused history taking, physical examination, and initiating diagnostic/consultation workflows), thereby emphasizing action-dependent information acquisition and structured decision-making.

**Role assignment**

**ED Attending:** Coordinate assessment & triage, make clinical decisions, organize standardized handoff. **Triage:** Confirm triage level, activate resuscitation, coordinate medical resources, exams & consultations. **ED Nurse:** Conduct monitoring, establish IV access, perform interventions, record key data & disease trends. **Family Communicator:** Verify medical history, explain clinical risks & benefits, soothe emotions, facilitate shared decision-making. **At-fault Driver (On-scene Narrator):** Provide biased on-scene info, guide learners to verify info & uphold professional boundaries.

**Key tasks**

Learners perform ABCDE rapid assessment with emphasis on airway risk, neurologic status and high-risk traumatic brain injury signs, establish clear division of labor and closed-loop teamwork, prioritize life support and determine the head CT pathway as well as triggers for consultation or monitoring escalation, verify key information and explain risks and benefits to support shared decision-making under pressure, and finally develop an appropriate disposition plan and complete structured handoff.

**Three decision checkpoints**

Throughout the simulation, learners progress through three sequential decision checkpoints that guide their clinical management. At each time-sensitive stage, they integrate real-time patient information, make critical clinical judgments, implement targeted interventions, and ensure effective communication and handoff to deliver timely and structured emergency care.

**At Checkpoint 1 (0–5 min)**, after documenting initial monitoring and ABCDE assessment, learners receive key information including visible head trauma, fluctuating vital signs, and drowsiness, then determine the triage level, activate the resuscitation workflow, and assign parallel tasks such as monitoring, IV access, hemostasis or immobilization, cervical spine precautions, and neurologic assessment.

**At Checkpoint 2 (5–15 min)**, after targeted questioning and repeated neurologic assessment, learners are informed of the patient’s clinical deterioration and inconsistent scene information, and must decide on life support escalation, imaging priority, consultation and monitoring triggers, and document discrepancies and reasoning.

**At Checkpoint 3 (15–30 min)**, after completing imaging orders, transport safety assessment and clinical summary, learners obtain imaging results and consultation feedback, then determine the disposition pathway, carry out structured handoff, and finish risk communication and collaborative decision-making.

**Expected outputs**

The documentation includes the initial triage and assessment summary with triage category, key neurologic findings and vital‑sign trends at corresponding time points, a decision log recording brief “decision–rationale–risk mitigation” notes for each checkpoint, a diagnostic, consultation and disposition plan involving priority ordering, triggers and handoff essentials, communication notes about verified key history, handling of inconsistent information and risk–benefit explanations, as well as a structured SBAR/ISBAR‑style handoff sheet for handover to senior physicians, specialty services or critical care teams.

**Debrief focus**

Debriefing emphasizes evidence-first reasoning and appraisal of information sources, prioritization and timeliness across the “triage–stabilize–diagnose–disposition/handoff” chain, closed-loop teamwork and documentation traceability, communication under pressure and boundary setting, and 2–3 transferable process improvements such as arrival checklist, consultation triggers, handoff template essentials.

### 2.3 Observation indicators

(1) *Theoretical and OSCE Scores:* The theoretical exam was designed by teaching physicians based on the resident training syllabus, covering knowledge of relevant diseases. It consisted of 50 questions, each worth 2 points, totaling 100 points. The exam was conducted in the computer lab of the hospital’s Education Office, lasting 1 h. It was administered online via the Educational Technology Platform, in group sessions, with strict proctoring by Education Office staff to prevent any form of cheating. If cheating was unavoidable, the student’s data was excluded. After the theoretical exam, practical skills were assessed uniformly using an OSCE-style multi-station examination in accordance with the National Standardized Resident Training Final Practical Skills Examination syllabus. Examiners were not members of the teaching group; all had participated in provincial practical skills examiner training and obtained qualification certificates (8). The total practical skills score was 100 points. The assessment took place in the skills training lab of the hospital’s Education Office. It involved on-site operations and group testing, with strict scoring according to skill evaluation standards, preventing any form of cheating. If cheating was unavoidable, the student’s data was excluded. Assessment scores ranged from 0 to 100; higher scores indicated better theoretical and practical teaching outcomes (9).
(2) *Teaching Satisfaction:* Physicians from different emergency departments evaluated the teaching process. The maximum score was 100 points. Scores of 80–100, 60–79, and <60 indicated “Very Satisfied,” “Satisfied,” and “Dissatisfied” respectively. Overall Satisfaction Rate = (Number of Satisfied + Number of Very Satisfied)/Total Number × 100%.
(3) *Recognition of the Teaching Process:* Survey content included: cultivation of thinking ability, communication ability, learning interest, knowledge understanding, and enhancement of practical application ability. The number of participants recognizing each aspect was counted, and the percentage was calculated.
(4) *Clinical Decision-Making Ability:* In simulated emergency scenarios and teaching cases, the accuracy of key decisions (e.g., triage accuracy, reasonableness of emergency management plans) was recorded for physicians in both groups.
(5) *Knowledge Retention Rate (Long-Term Effect Evaluation):* At a specified time (3 months) after the end of teaching, physicians in both groups (unaware of the test beforehand) underwent the same or similar theoretical knowledge and practical tests, following the same model as (1). The smaller the decline in each score from the baseline value, the higher the knowledge retention rate was considered.

### 2.4 Statistical processing

Categorical variables are presented as *n* (%). Continuous variables are presented as mean ± standard deviation (SD) when approximately normally distributed; otherwise, they are presented as median (interquartile range, IQR). Between-group comparisons used the chi-square test or Fisher’s exact test for categorical variables, the independent-samples *t*-test for normally distributed continuous variables, and the Mann–Whitney U test for non-normally distributed continuous variables. All tests were two-tailed, with statistical significance set at *p* < 0.05. Analyses were performed using SPSS version 22.0 (SPSS Inc., Chicago, IL, United States).

#### 2.4.1 Sample size justification

This study used a pragmatic, feasibility-based sample size, determined by the number of residents rotating through the emergency department during the study period (November 2024 to March 2025). No *a priori* sample size calculation was performed, consistent with the exploratory nature of this educational intervention. As such, the findings should be interpreted as preliminary, and future multicenter studies with formal power calculations are warranted (Table 1).

**Table 1:** Baseline characteristic comparison (by batch/group allocation).

Characteristic	Control group (*n* = 18)	Observation group (*n* = 18)	Inter-group difference (95% CI)	*p*-value
Sex [*n* (%)]				
Male	8 (44.4)	9 (50.0)	−5.6% (−35.7, 24.5)	0.747
Female	10 (55.6)	9 (50.0)	5.6% (−24.5, 35.7)	0.747
Age (years)				
Mean ± SD	25.71 ± 2.50	25.68 ± 2.47	0.03 (−1.76, 1.82)	0.975
Range	23–33	24–32	–	–
Cohort/batch, *n* (%)				
Batch 1	9 (50.0)	9 (50.0)	0.0% (−30.1, 30.1)	1.000
Batch 2	9 (50.0)	9 (50.0)	0.0% (−30.1, 30.1)	1.000

Baseline data collection and before the start of instruction, all variables between the two groups were compared using independent samples *t*-test (continuous variables) or chi-square test (categorical variables). Allocation was performed by batch/cohort, with the same teaching method used within the same batch to avoid contamination. All baseline data collection was complete and without missing values.

#### 2.4.2 Cluster allocation considerations

To minimize contamination between intervention and control groups, allocation was performed at the cohort level (i.e., all residents within the same rotation cohort received the same teaching method). Although outcomes were analyzed at the individual level, we did not apply cluster-robust variance estimation due to the small number of cohorts (*n* = 2 per group), which may lead to underestimation of standard errors and narrower confidence intervals. To assess the robustness of our findings, we conducted a sensitivity analysis using cohort-level aggregated data for the primary outcomes, which yielded consistent effect directions and magnitudes (see Supplementary Table S1). This supports the conclusion that the observed effects are not solely attributable to clustering artifacts.

To assess the robustness of primary findings to missing follow-up data, we planned best-case and worst-case sensitivity analyses for the 3-month knowledge retention outcomes. In the best-case scenario, the missing participant was assigned the highest observed score in the control group; in the worst-case scenario, the lowest observed score was imputed. These analyses were performed for both theoretical and OSCE score declines.

#### 2.4.3 Blinding

Due to the nature of the educational intervention, blinding of participants and instructors was not feasible. However, OSCE assessors were independent of the teaching team and blinded to group allocation. All OSCE stations used standardized checklists and scoring rubrics, and assessors underwent uniform training prior to the examination to ensure consistency and reduce performance bias.

## 3 Results

### 3.1 Comparison of theoretical and OSCE scores between control and observation groups

**Figure 3:**
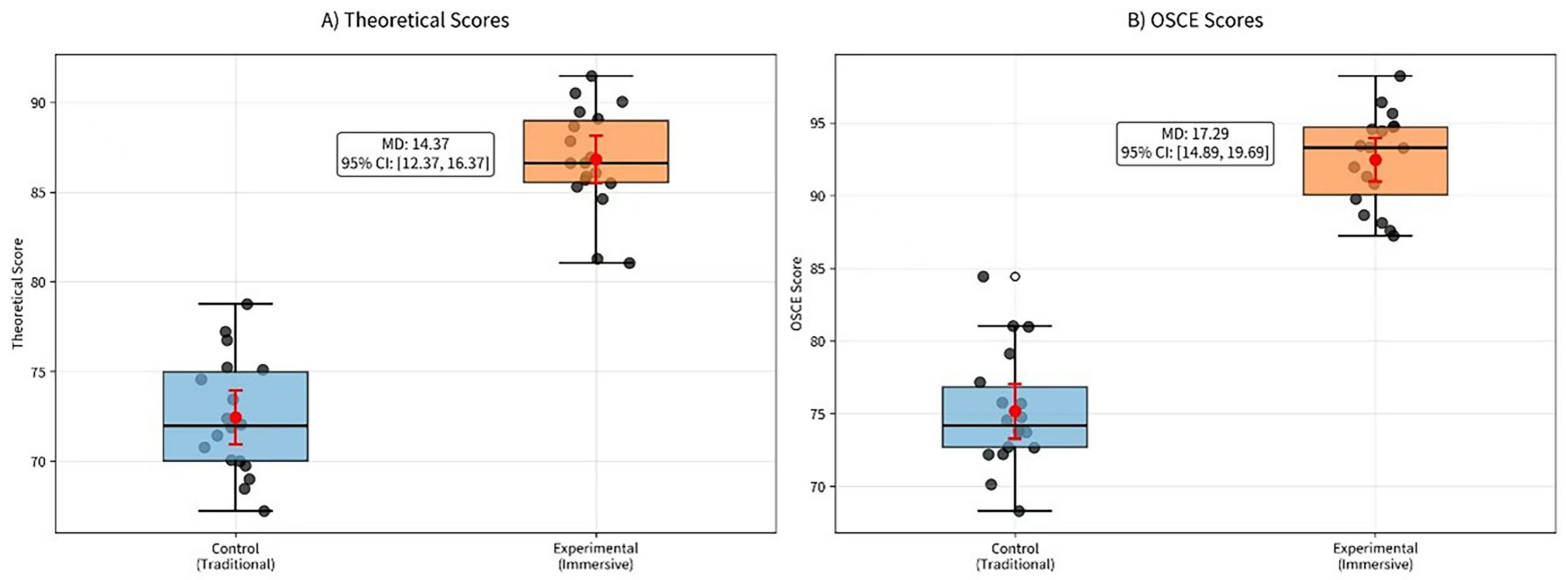
Theoretical and OSCE assessment scores after a 3-month teaching period. **(A)** Theoretical examination scores. **(B)** OSCE scores. Points represent individual trainees’ scores. MD denotes the between-group mean difference (experimental minus control), and 95% CI denotes the 95% confidence interval. Red dots indicate group means, and red error bars represent mean ± SD.

The theoretical assessment scores and OSCE scores of the observation group were significantly higher than those of the control group (*p* < 0.001) (see Table 2; Figure 3).

**Table 2:** Comparison of theoretical and OSCE scores for physicians in the control group and observation group (points, x ± s).

Group	Theoretical assessment (Mean ± SD)	OSCE score (Mean ± SD)
Control group (*n* = 18)	72.45 ± 3.25	75.18 ± 4.08
Observation group (*n* = 18)	86.82 ± 2.85	92.47 ± 3.22
Inter-group difference MD	14.37 (12.37, 16.37)	17.29 (14.89, 19.69)
*P*-value	<0.001	<0.001

SD, standard deviation.

### 3.2 Comparison of teaching satisfaction and recognition of the teaching process and clinical decision-making ability between control and observation groups

**Figure 4:**
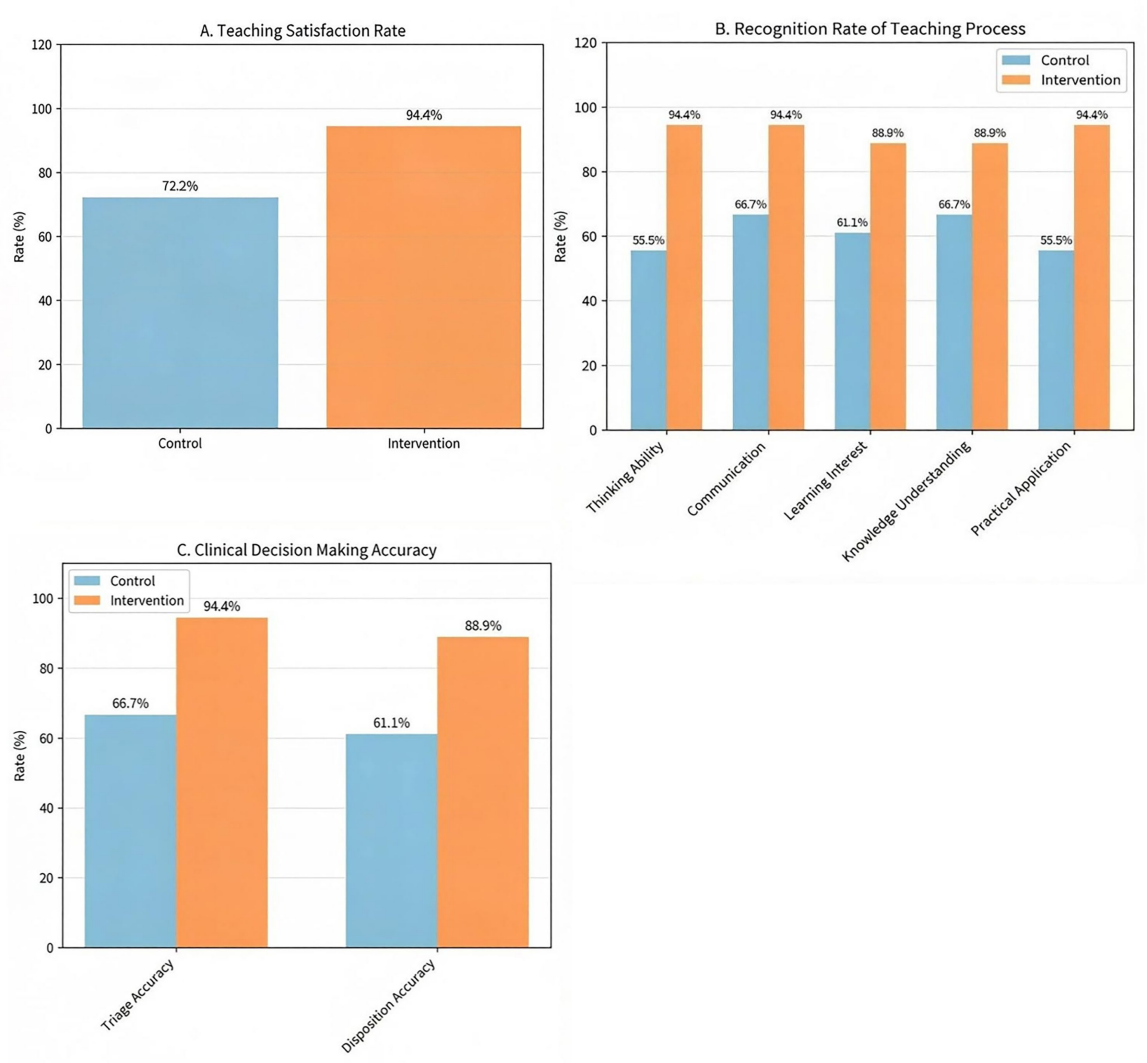
Cohort-allocated comparison between the intervention and control groups. Bars indicate group means. **(A–C)** Show higher teaching satisfaction, stronger recognition of the teaching process, and better clinical decision-making indicators in the intervention group than in the control group.

Teaching satisfaction in the observation group was significantly higher than in the control group (*p* < 0.05). The observation group showed significantly higher recognition than the control group with statistically significant improvements in thinking ability, communication skills, and practical application (*p* < 0.05), while learning interest and knowledge understanding showed numerical improvements that did not reach statistical significance. The triage accuracy rate in the observation group was significantly higher than that in the control group (*p* = 0.034), and the intervention group showed a higher proportion of reasonable emergency management plans compared to controls (88.89% vs. 61.11%), with a risk difference of 27.8% (95% CI: −0.5 to 56.1%; *p* = 0.054) (see Table 3; Figure 4).

**Table 3:** Comparison of teaching satisfaction and recognition of the teaching process and clinical decision-making ability between control and observation groups [cases].

Survey items	Control group (*n* = 18)	Observation group (*n* = 18)	RD (95% CI)	*p*-value
Overall satisfaction, *n* (%)	13 (72.2)	17 (94.4)	22.2% (−0.8,45.2)	0.034
Recognition of the teaching process				
Ability of thinking	10 (55.6)	17 (94.4)	38.9% (13.6,64.2)	0.007
Communication skills	12 (66.7)	17 (94.4)	27.8% (2.1,53.5)	0.034
Learning interest	11 (61.1)	16 (88.9)	27.8% (−0.5,56.1)	0.054
Knowledge understanding	12 (66.7)	16 (88.9)	22.2% (−5.8,50.2)	0.109
Practical application	10 (55.6)	17 (94.4)	38.9% (13.6,64.2)	0.007
Clinical decision-making indicators [*n* (%)]				
Accurate triage	12 (66.7)	17 (94.4)	27.7% (2.0,53.4)	0.034
Reasonable management plan	11 (61.1)	16 (88.9)	27.8% (−0.5,56.1)	0.054

Post-treatment questionnaire results are expressed as *n* (%), with intergroup comparisons using the risk difference (RD) (95% CI). Agreement is defined as the proportion of responses “yes/agree.”

### 3.3 Comparison of knowledge retention rate between control and observation groups

The knowledge retention rate in the observation group was significantly higher than in the control group (*p* < 0.001) (see Table 4).

**Table 4:** Comparison of knowledge retention rate after teaching between control and observation groups (points, x̄±s).

Group	Theoretical examination (Mean ±SD)	OSCE (Mean ±SD)	Theoretical decline from baseline*	OSCE decline from baseline*
Control group (*n* = 17)	59.85 ± 4.95	62.12 ± 5.92	12.60	13.06
Observation group (*n* = 18)	79.03 ± 3.90	83.35 ± 4.58	7.79	9.12
Inter-group difference (MD)	19.18 (16.22, 22.14)	21.23 (17.71, 24.75)	–	–
*p* value	<0.001	<0.001	–	–

SD, standard deviation. * One participant in the control group was lost to follow-up at the 3-month assessment. Score decline was calculated as (baseline score − follow-up score); a smaller decline indicates better knowledge retention. Baseline scores are shown in Table 2. Best-case and worst-case sensitivity analyses for the missing follow-up assessment yielded unchanged between-group interpretation (*p* < 0.001; see Supplementary Table S2).

## 4 Discussion

### 4.1 “Script Killing” immersive teaching system systematically breaks through the three major bottlenecks of traditional emergency teaching

In this cohort-allocated controlled educational study, we found that “Script Killing” immersive teaching was associated with improved competency among residents rotating through the emergency department across departments. Traditional teaching often leads to insufficient training in multitasking abilities due to fragmented scenarios (e.g., the synchronization of cardiopulmonary resuscitation and communication with dependents is difficult to simulate). The “Script Killing” integrates fragmented clinical scenarios into a coherent task chain through the whole process simulation of “pre-hospital triage—in-hospital rescue—ward handover,” enabling physicians to practice emergency response capabilities in a highly simulated pressure environment (triage accuracy rate in the observation group was 94.44%; rationality rate of treatment plans was 88.89%). Role responsibility binding mechanisms (such as clearly defining the responsibilities of “rescue commander” and “communication specialist”) effectively avoid the “diffusion of responsibility effect” in team collaboration. The improvement in the OSCE practical skills scores of the observation group validates that well-defined roles enhance interprofessional collaboration. More critically, the NPC dynamic intervention mechanism (such as simulated dependents refusing to sign, sudden drug allergy, and other conflicts) provides a “safe sandbox” for zero-risk trial and error, enabling in-depth training for high-risk operations and compensating for the lack of error tolerance in traditional teaching (10). Although the improvement in the reasonableness of emergency management plans did not reach statistical significance (*p* = 0.054), the effect size (RD = 27.8%) suggests a clinically meaningful trend. The lack of significance may reflect a type II error due to the limited sample size (*n* = 18 per group), as this study was pragmatically sized based on available residents rather than a formal power calculation. Future multicenter studies with larger cohorts are needed to confirm this potential benefit.

### 4.2 “Script Killing” immersive teaching achieves dual upgrade of learning motivation and clinical behavior

The high immersion design of “Script Killing” (such as countdown rescue, resource constraints, and other variables) was associated with higher learner engagement and a numerical increase in reported learning interest. The recognition rate of learning interest in the observation group reached 88.89%, far exceeding the 61.11% of the control group. Role-playing forces physicians to shift from “passive listeners” to “decision-makers.” For example, when an internist plays the role of an emergency attending physician, they need to break through the barriers of specialized thinking and quickly formulate a rescue plan. This process is in line with the “experience-association” principle of adult learning, prompting the autonomous construction of a knowledge framework. The closed-loop review mechanism of “role experience → immediate feedback → strategy correction” accelerates the transformation of knowledge into behavior. For example, after simulating the scenario of “Dependents’ emotional breakdown”, the path of “learning by doing and correcting mistakes” through targeted feedback from NPCs and group reflection explained the internal logic of the synchronous improvement of teaching satisfaction (94.44% vs. 72.22%) and assessment scores (11).

### 4.3 Reconstruction of ability culture mode of immersive teaching of “Script Killing”

For standardized residency training, SKIT reconstructs the ability Culture model, which has revolutionary significance. Compared to traditional scenario simulation, its advantages are reflected in breakthroughs in three dimensions: Firstly, Resource allocation and triage strategies are trained through composite scenario design (such as simultaneously managing cranioencephalic trauma and emergency obstetric care in “multi-vehicle cap collision”), which solves the limitations of single-case teaching. Secondly, role difficulty grading (entry-level nurse → advanced commander) matches the needs of physicians with different years of experience to avoid the coverage deviation of “one-size-fits-all” teaching. Thirdly, integrate Objective Structured Clinical Examination (OSCE) and behavioral observation (team coordination effectiveness) into a multi-dimensional evaluation system to comprehensively quantify the competency improvement effect (12).

### 4.4 Positioning of SKIT among simulation-based immersive teaching modalities in emergency medicine education

Compared with High-Fidelity Simulation (HFS), which depends on advanced manikins and dedicated simulation centers and is limited by high costs and poor scalability for frequent training, SKIT boasts better cost-effectiveness, accessibility and scalability, as it only needs narrative scripts and role-play and can be implemented in ordinary spaces without specialized equipment. Yet HFS outperforms SKIT in training fine motor procedural skills like intubation and central line placement, while SKIT excels at fostering non-technical skills (NTS) such as interprofessional teamwork, pressure communication and dynamic clinical decision-making—vital Emergency Medicine (EM) competencies often overlooked in HFS curricula centered on technical proficiency.

SKIT and Virtual Reality (VR) both deliver immersive learning but differ greatly in social interaction and resource requirements. VR offers unrivaled individual visual–spatial immersion for standardized repetitive training yet demands high-end headsets, isolates learners and restricts real-time interdisciplinary communication, while also posing technical barriers and motion sickness risks. In contrast, SKIT is an inherently social, interactive modality centered on face-to-face communication among learners, closely mirroring the communicative, dynamic nature of real emergency department workflows, with no such technical or physical drawbacks.

## 5 Conclusion

This study confirms that the Script Killing Immersive Teaching (SKIT) model, through the construction of a full-process task chain (pre-hospital-in-hospital-ward full cycle), role responsibility binding (clear division of labor to avoid bystander effect), and dynamic variable intervention (NPC creates real conflicts) three core mechanisms, significantly improves the clinical competence of cross-departmental rotating physicians. Its value is not only reflected in the quantitative improvement of theoretical assessment and OSCE scores (*p* < 0.001), but also in breaking through the mindset barriers of non-emergency specialists and strengthening key capabilities such as emergency management and interprofessional collaboration. This teaching method significantly improved the teaching satisfaction and learning initiative of resident physicians, and had better long-term knowledge retention effects, providing a roll-out innovative solution to address the low competency attainment rate of emergency residency training positions.

This study has several limitations. As a single-center pilot with only 18 participants per group, its generalizability is limited. Cohort allocation (non-randomization) and the lack of blinding of participants and instructors may have introduced performance bias. Additionally, cluster-robust variance estimation was not used due to few clusters, potentially affecting statistical precision. Only short-term outcomes were measured, and the SKIT framework currently does not address technical procedural skills, which warrants future hybrid integration with high-fidelity simulation. In addition, the comprehensive roll-out still needs to address three major challenges: First, the need for scenario standardization requires the establishment of a scenario library covering typical emergency scenarios such as poisoning, trauma, and cardiopulmonary resuscitation, and incorporation into the mandatory residency training curriculum; Second, there is a bottleneck in the teaching staff’s capabilities. Instructors need to master script design, dynamic intervention, and debriefing guidance skills. It is recommended to carry out special certification training. Third, the space for technology integration, which can be combined with VR technology to simulate epigastric upset rescue environment to enhance immersion (13), and use AI to analyze students’ decision-making paths to adaptively adjust the difficulty of the scenario. It can be further extended to fields with high collaboration demands such as critical care medicine and disaster relief, promoting the upgrading of medical education from “skills training” to “clinical thinking reshaping” (14, 15).

## Data Availability

The original contributions presented in the study are included in the article/Supplementary material, further inquiries can be directed to the corresponding author.
