# Supplementary material for: “Script Killing” immersive teaching improves the competency of emergency medicine residents rotating across departments
Source: Front Public Health. 2026 Mar 19;14:1806065. doi: 10.3389/fpubh.2026.1806065 (PMC13044127; doi:10.3389/fpubh.2026.1806065)
Supplement: Supplementary file 1 [file supplementary_file_1.docx]

***Supplementary Table S1***

**Cohort-Level Sensitivity Analysis for Primary Outcomes**

| Outcome | Control Cohort (n=2) | Intervention Cohort (n=2) | Effect Size (MD) |
| --- | --- | --- | --- |
| Theoretical Score (mean) | 72.45 | 86.82 | 14.37 |
| OSCE Score (mean) | 75.18 | 92.47 | 17.29 |

Note: Cohort-level means were calculated from individual-level data. Due to the small number of cohorts, inferential statistics were not applied; results are presented descriptively to support individual-level findings.

***Supplementary Table S2***

**Best-Case and Worst-Case Sensitivity Analysis for 3-Month Knowledge Retention**

| Outcome | Scenario | Control Group (n=18)  Mean±SD | Observation Group (n=18) Mean±SD | MD  (95% CI) | P-value |
| --- | --- | --- | --- | --- | --- |
| Theoretical Decline | Best-case | 11.9±4.50 | 7.79±3.90 | 4.19(1.38,7.00) | 0.004 |
|  | Worst-case | 13.22±5.10 | 7.79±3.90 | 5.43(2.40,8.46) | 0.001 |
| OSCE Decline | Best-case | 12.50±5.20 | 9.12±4.58 | 3.38(0.10,6.66) | 0.043 |
|  | Worst-case | 14.10±5.80 | 9.12±4.58 | 4.98(1.50,8.46) | 0.006 |

Note:MD, mean difference; CI, confidence interval. Best-case scenario imputed the highest observed score in the control group for the missing participant; worst-case scenario imputed the lowest observed score. All values are presented as mean ± standard deviation.
